# A systematic review of participatory approaches to empower health workers in low- and middle-income countries, highlighting Health Workers for Change

**DOI:** 10.1093/inthealth/ihac070

**Published:** 2022-11-09

**Authors:** Obaraboye Olude, Carol Vlassoff, Julienne Niyikora, Alison Krentel

**Affiliations:** School of Epidemiology and Public Health, University of Ottawa, Ottawa K1G 5Z3, Canada; School of Epidemiology and Public Health, University of Ottawa, Ottawa K1G 5Z3, Canada; Bruyère Research Institute, Ottawa K1N 5C8, Canada; Bruyère Research Institute, Ottawa K1N 5C8, Canada; School of Epidemiology and Public Health, University of Ottawa, Ottawa K1G 5Z3, Canada; Bruyère Research Institute, Ottawa K1N 5C8, Canada

**Keywords:** empowerment, health workers, LMICs, motivation, participatory approaches, positive change

## Abstract

This systematic review assesses participatory approaches to motivating positive change among health workers in low- and middle-income countries (LMICs). The mistreatment of clients at health centres has been extensively documented, causing stress among clients, health complications and even avoidance of health centres altogether. Health workers, too, face challenges, including medicine shortages, task shifting, inadequate training and a lack of managerial support. Solutions are urgently needed to realise global commitments to quality primary healthcare, country ownership and universal health coverage. This review searched 1243 titles and abstracts, of which 32 were extracted for full text review using a published critical assessment tool. Eight papers were retained for final review, all using a single methodology, ‘Health Workers for Change’ (HWFC). The intervention was adapted to diverse geographical and health settings. Nine indicators from the included studies were assessed, eliciting many common findings and documenting an overall positive impact of the HWFC approach. Health workers acknowledged their negative behaviour towards clients, often as a way of coping with their own unmet needs. In most settings they developed action plans to address these issues. Recommendations are made on mainstreaming HWFC into health systems in LMICs and its potential application to alleviating stress and burnout from COVID-19.

## Introduction

Goal 3 of the Sustainable Development Goals^1^ adopted by the United Nations in 2015 included the target of universal health coverage by 2030, encompassing financial risk protection, access to quality essential healthcare services, access to safe, effective, quality and affordable essential medicines and vaccines for all. The definition of universal health includes both ‘universal access to health’ and ‘universal health coverage’, which ‘imply that all people and communities have access, without any kind of discrimination, to comprehensive, appropriate and timely, quality health services’.^2^ Achieving this target entails greater efficiency and effectiveness in the health services, their greater responsiveness to community needs and an organisational culture of client service imbued with quality and equity.^3^ It also means that health workers, the main agents of institutional transformation, have the training, will, working conditions and tools to embrace and promote this vision.^4^

Research has documented numerous barriers to quality healthcare in low- and middle-income countries (LMICs),^3–5^ including insensitive treatment of clients at health centres. Client reports of offensive treatment by health workers include blaming for late attendance at the facility or not following given instructions, sometimes resulting in health complications or even avoidance of health centres altogether.^3–6^ Health workers, on the other hand, face numerous challenges at work, from lack of tools, medicines and equipment to inadequate training, supervision and supervisory support.^3,4,7^ This can trigger negative coping behaviour, such as absences from work, corruption, lack of engagement and poor productivity.^8^

Despite the widespread recognition of health worker motivational deficiencies, ways of addressing them have received less attention in the literature.^4^ For example, a large systematic review of policy interventions to improve health worker performance, including in LMICs, failed to include health workers’ motivation,^9^ perhaps due to the few studies on this topic.^3^ A more recent systematic review of human resource management policies in LMICs, including supervision, compensation, systems support and lifelong learning interventions, showed limited impact on the motivation of physicians and nurses^3^ in the contexts in which they were introduced, although supportive supervision was a positive feature in health workers’ reported satisfaction.

In our search for successful methods in motivating positive behavioural change in LMICs we considered that interventions, such as those reviewed above, which were largely institutional in nature, might have proven more successful if developed in a participatory manner with health workers themselves, given that their own suggestions and experiences are part of the desired changes. We also considered that interventions involving whole health teams, rather than only higher-level personnel, could enrich these interventions, as their work is interdependent and collaborative. The authors were already familiar with literature on one such participatory intervention based on the methodology of Paulo Freire, ‘Health Workers for Change’ (HWFC), which, to our knowledge, has not been systematically reviewed. We therefore included it in our literature search, as well as other participatory interventions focusing on motivating change among health workers in LMICs. We limited our search to participatory interventions involving problems and solutions posed by health teams themselves, rather than didactic interventions, such as policies, guidelines and handbooks, designed to transfer information or skills to them. There is growing evidence that the latter tools, which are resource-intensive to develop,^7^ are rarely used in the settings for which they are intended.^7,10^

Paulo Freire's concept of participatory approaches^9^ argues that change must actively involve the people for whom it is intended, rather than being imposed from outside. It stresses the development of critical consciousness in learners and building their confidence in their own capacity to solve problems. In his participatory research methodology, the research subjects or learners are motivated via the intervention to identify challenges and solutions. From its inception in the 1970s, Freire's approach^11^ has inspired generations of educators seeking to transform didactic training into dynamic learning for action.^12–14^ While it has remained popular in the educational sector, Freire's approach has been adopted less frequently in the public health domain and mainly in the areas of health promotion,^15,16^ treatment of selected health conditions,^17,18^ and the analysis of health curricula, such as nursing^19^ and health education.^20^ A fundamental aspect of this approach is that the participants themselves identify problems and solutions, while the researchers documenting it are independent of the process.^3^ This is distinct from participatory action research or community-based participatory research, in which the researchers are partners in the process.

Our research question therefore asked: What participatory methods exist that involve health workers themselves in identifying challenges and potential solutions at work, and to what extent have they generated positive changes?

## Methodology

The protocol for this systematic review was registered with the International Prospective Register of Systematic Reviews (PROSPERO, CRD42021261050) in June 2021.

### Inclusion criteria

Our literature search was conducted from April to July 2021, with the following inclusion criteria:

- Language: published articles in English, French and Spanish.
- Location: LMIC focus^1^.
- Research type: original peer-reviewed research.
- Methods and target group: participatory empowerment methods for health workers in LMIC settings, based on Freire's concept (above), and where researchers were independent from the identification of challenges and solutions.^13^
- Results: adequately described some of the impact indicators.
- Impact: effectiveness in motivating positive change evaluated in indicators reported in the studies.

French and Spanish sources were considered because HWFC had been piloted or tested in at least two French and Spanish-speaking countries and the review team had the requisite language capacity.

### Search strategy

The University of Ottawa Library's search tool (OMNI) and Google databases were searched for the period January 1998 to May 2021^2^. OMNI searches the University of Ottawa's print collection and different databases it subscribes to, including PubMed (Medline), SCOPUS, Web of Science, APA PsycInfo (OVID), Medline (OVID), the University's Research Institutional Repository and 13 other Ontario university libraries.

English keywords included the following (the most frequently encountered in our search are in bold, and a backslash between keywords or phrases indicates that both were searched): health workers’ manual; community health workers’ manual; method* for motivat* health workers/community health workers; method*/manuals to **help** health workers//community health workers **address problems/improve quality of care** /**improve** health worker- /community health worker- **client relations; change management** of health workers; motivat* change among health workers/community health workers; e**mpowering** health workers/community health workers; **supporting/supportive interventions** for health workers/community health workers; and methodology/methods/manuals to **empower/support** health workers//community health workers. The same keywords were translated for the French and Spanish sources and the same databases were employed. These search terms were used in the OMNI search advanced setting to include subject, titles and abstracts. Identical keywords were used for the PubMed and Google searches. A snowball process (checking references of included articles) was conducted by the two first authors (OO, CV) for other sources that might have been overlooked. Books and non-peer reviewed sources were excluded.

While not included in our search criteria, we consulted grey sources^3^,^21–23^ literature from high-income countries^14–16^ and studies focusing on specific health conditions, such as maternal and child health, as we encountered them, so as not to miss any relevant participatory approaches.

### Eligibility and quality assessment

**Figure 1.**
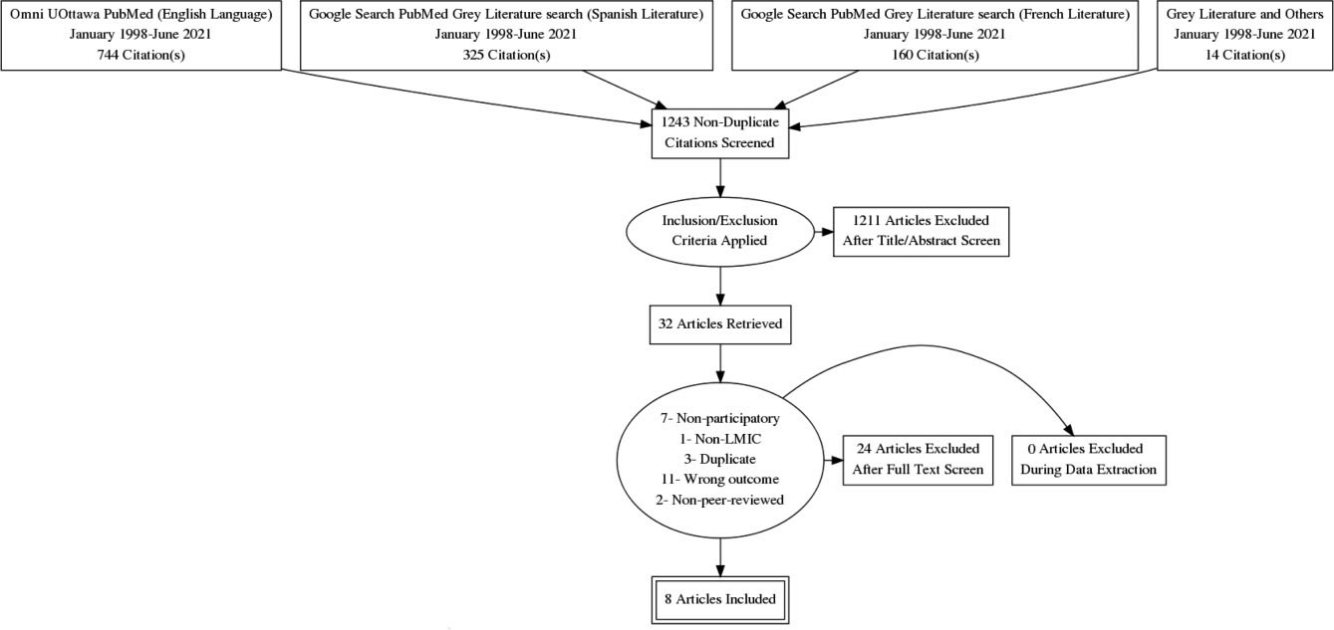
The PRISMA flowchart shows data sources and search results: 1243 titles resulted from the initial search. Following title/abstract review, 32 articles were selected for full review. An additional 24 articles were excluded, leaving 8 included in the analysis.

The PRISMA flowchart is presented in Figure 1. Our initial search yielded 1243 titles: English (758), French (160) and Spanish (325). These articles were assessed for relevance through title scan and abstract review. Abstracts of papers meeting our inclusion criteria were independently reviewed by two authors, OO and CV, and 32 papers were selected for full text review if at least one author had reasons to feel they warranted review by a second author. Thirty-one papers were independently assessed by OO and CV, and one other, on which CV was a co-author, was reviewed by JN and OO to avoid any conflict of interest.

### Data extraction

The 32 selected papers were interrogated using a published critical appraisal tool with a validated scoring system^4^ for systematic reviews, applicable to both qualitative^24–26^ and quantitative data^27^ (Table 1). The form facilitated a more rigorous review of the articles and their appropriateness for addressing our research question. The results led us to exclude 24 studies, 14 because no impact evaluation was done, 4 because they were conducted in high-income countries, 3 because the interventions were non-participatory and 3 because they were not peer-reviewed^5^.

**Table 1.** Criteria for evaluation of papers

Paper section	Good	Fair	Poor	Very poor
Introduction/objectives: Background and clear statement of research aims?	-Full but concise background -Up-to-date literature review highlighting knowledge gaps -Clear statement objectives including research questions	-Some background and literature review -Research questions outlined.	-Some background but no objectives/questions, OR -Objectives but inadequate background	-No mention of aims/objectives, background or literature review
Methods/data: Is the method appropriate and clearly explained?	-Method appropriate and clearly described (e.g. questionnaires included) -Details of data collection and recording	-Method appropriate, description missing information -Data described	-Questionable if method is appropriate -Method described inadequately -Little data description	-No mention of method, AND/OR -Method inappropriate, AND/OR -No f data description
Sampling: Sampling strategy appropriate to meet objectives	-Details of sample population (age/gender/race/context) and how recruited -Why group(s) selected -Sample size justified and appropriate for study -Response rates shown and explained	-Sample size justified -Most necessary information given, but some missing	-Sampling mentioned but few descriptive details	-No sample details
Data analysis:	-Clear description of how analysis done Qualitative studies: -Description of how themes derived/ respondent validation or triangulation Quantitative studies: -Hypothesis driven -Calculations correct/statistical significance discussed	Qualitative: -Descriptive discussion of analysis Quantitative -Methods incompletely described	-Minimal details about analysis	-No discussion of analysis
Ethics and bias: Ethical issues addressed and necessary ethical approval obtained? Researcher–participant relationships considered?	Ethics: -Where necessary, issues of confidentiality, sensitivity and consent addressed Bias: -Researcher reflective and/or aware of own bias	Ethics/bias issues Acknowledged but insufficiently addressed	-Brief mention of issues	-No mention of issues
Findings/results: Clear statement of findings?	-Findings explicit, easy to understand, in logical progression -Tables, if present, explained in text -Results relate directly to objectives -Sufficient data presented to support findings	-Findings incompletely explained -Data presented relate directly to results	-Findings presented haphazardly, not explained in detail	-Findings not mentioned or do not relate to objectives
Transferability/generalisability: Findings transferable (generalisable) to a wider population or potentially other settings?	-Study context and setting described sufficiently to permit Comparison with other contexts, PLUS high score on Item 3 (sampling)	-Some context and setting described, but more needed to replicate/compare with other studies PLUS fair score or higher in Item 3	-Minimal description of context/setting	-No description of context/setting
Implications and usefulness: Importance of findings to policy and/or practice?	(1) Contributes new understanding, insight, perspective (2) Suggests ideas for further research (3) Suggests implications for policy and/or practice	-2 of the 3 elements in column 2 scored ‘good’	-Only 1 of elements scored ‘good’	-None of elements scored ‘good’

Scoring—Good=4, Fair=3, Poor=2, Very Poor=1 (possible total of 32).

*Slightly adapted from Hawker et al. (2002).^1^

## Results

### Study selection and characteristics

Eight articles that met our criteria (Table 1) were selected for full review. Only one approach, ‘Health Workers for Change’, had the requisite evidence to fully address our research question on proven approaches for motivating positive change in health workers’ behaviour in LMIC settings using a participatory approach. Gender was a focus of all studies, but the participatory approach elicited a range of more general concerns about quality of care (QoC) and other problems within the health services. Our analysis is therefore not limited to gender outcomes alone but focuses on the findings concerning the health system as a whole.

### Health workers for change methodology

‘Health Workers for Change’ (HWFC) was developed by the Women's Health Project^6^ in South Africa and based on Freire's participatory approach of open communication and dialogue, in which participants in the process identify and elaborate upon various aspects of their role and experiences as health workers. The HWFC methodology consists of a series of six 2–3-h workshops integrated into regular health facility activities and implemented by facilitators trained to draw out the experiences of the health worker participants. In keeping with Freire's principle of participant-led problem posing and exploration, motivated by trained facilitators who were trained to draw out the health workers’ responses, as opposed to suggesting answers or directing the participants toward particular observations or conclusions. Similarly, the researchers did not participate in the workshops but rather observed and documented the process, including information about the different products generated (e.g. participants’ depictions of their ‘river of life’, contributions such as poems or stories and action plans). The first five workshops explore reasons why health workers chose their profession, their relationships with other staff and clients, gender issues, unmet needs and overcoming obstacles at work. The sixth and final workshop focuses on developing solutions in the form of action plans developed as a result of the challenges and potential solutions identified in the workshops. This original format was modified to meet specific needs and circumstances in the different studies. The included papers focused on: developing the approach in rural South Africa,^28^ its testing for acceptability in four other African countries (Zambia, Mozambique, Uganda and Senegal),^29^ its further testing for impact in Kenya^30^ and Argentina,^31^ a multicountry comparison in seven countries (Nigeria [two sites], Tanzania [two sites], Ghana, Kenya and Argentina)^32^ and three other studies, one in Pakistan^33^ and two in Tanzania.^24,25^

### Critical appraisal of included studies

The first two studies conducted in South Africa^28,29^ were exploratory, using qualitative methods. The next three studies, in Kenya,^30^ Argentina^31^ and the multicountry study,^32^ used similar designs based on a common research protocol, although their methods varied somewhat according to individual context. All of these studies used a longitudinal design to measure change over three time periods and included the triangulation of several different methods: qualitative methods (key informant interviews, group interviews, focus groups) and quantitative instruments (structured observations, time flow observations, individual interviews, records reviews). The sixth study, in Pakistan,^33^ used qualitative methods, mainly participatory observation and synthesis by the study team. The seventh study, in Tanzania,^34^ used baseline and postintervention questionnaires to evaluate the impact of the intervention, while the eighth^35^ used questionnaire surveys and focus group discussions. Table 2 includes details about the design, sample and setting, research focus, results, implications and weaknesses of these studies. Several of the studies included more than one study location, or respondents from different locations who attended the workshops in a nearby facility.

**Table 2.** Selected details of included studies

Title, year, authors	Design, sample, setting	Research focus	Results	Implications	Weaknesses	Score Based on Table 1 criteria
Fonn and Xaba (2001).^29^ Health Workers for Change: developing the initiative	Qualitative, 6 workshops in a rural South African clinic All clinic staff (16), participated	Development of HWFC methodology	Self-analysis and recognition of need to improve their QoC. HW saw their services as uncaring and punitive. Resulting in commitment to change (e.g. regular staff meetings, conflict resolution workshop and strategy for relating to superiors)	Generated information for further development. Showed it was possible to develop a method to explore sensitive issues and generate solutions. Importance of well-trained facilitators emphasised	Analysis approach not described Impact on client relations not mentioned	22
Fonn et al. (2001).^30^ Health providers’ opinions on provider–client relations: results of a multicountry study to test Health Workers for Change	Qualitative, 4 countries (Zambia, Mozambique, Uganda and Senegal). Same protocol used to minimise variation	To test the acceptability and effectiveness of HWFC in 4 African countries	Similar QoC inadequacies identified, (drugs shortages, poor working conditions, late salaries) and sensitive issues (bribery, poor treatment of women) with suggestions to improve	Indicates that poor QoC demands broader response to address systemic issues. Intervention found acceptable in different situations, generalisable and a good change management tool	Inadequate information on data collection and analysis provided	25
Onyango-Ouma et al. (2001).^31^ The Health Workers for Change impact study in Kenya	Triangulation of methods; qualitative (KIIs, FGDs) and quantitative (questionnaires, observations) to measure changes over 3 time (T) points. Data collected from clients, HW in facility and managers	Impact of HWFC intervention in rural Kenya on QoC provided	Changes in 5 indicators of QoC: (1) client waiting time: reduced over 3 Ts (2) HW–client communication: clients found providers more polite at T2 and T3 (3) Provider attitudes improved participants (4) Service provision: clients noted more drugs available at T2 and T3 (5) Clients’ opinions about HW: clients reported positive changes in some HWs	Method used adds strength to findings To increase impact of workshops, authors suggest: (1) implement annually (2) Involve district-level managers (3) Conduct only in an open responsive system to facilitate longer term impact	Analysis methods should be more fully explained and ethical clearance procedures not presented	26
Pittman et al. (2001).^32^ An assessment of the impact of Health Workers for Change in Avellaneda, Province of Buenos Aires, Argentina	Triangulation of methods; qualitative (KII, FGD, group interviews) and quantitative (questionnaires) in two poor urban clinics (one study, one control) at 3 time (T) points Collected data from community and HW in facility and system levels	Impact of HWFC intervention on QoC in a poor urban clinic in Argentina	Changes in 6 areas of QoC: 1) Increased desire and ability to affect change in QoC from T1-T2 (2) Perceived need to improve attitudes toward clients—some positive change in how staff treated clients (3) Gender sensitivity positively increased (4) Relations between HW deteriorated (5) Attitudes towards authorities –frustration with facility director (6) Commencement of staff meetings for a period	Method used adds strength to findings Difficult to separate impact of HWFC from ongoing interventions in area Lasting change was inhibited by unresponsive director Suggested district-wide intervention	Methodology not well described Differences between study and control facility not systematically presented Participants’ characteristics not shown	25
Shaikh et al. (2006).^34^ Health Workers for Change: a tool for promoting behaviour change among health providers	Qualitative, in 7 field sites, both urban and rural, in all 4 provinces and the Federally Administered Northern Areas of Pakistan	Evaluate the HWFC methodology in the Pakistani context	Detailed results (e.g. motivations for becoming HW) Women's inferior social status recognized Obstacles to QoC: logistical, managerial, social and behavioural Dignity and recognition more important incentives than salary for HWs Need for change recognised and practical action plans developed	Reorientation and strengthening of health system, improved client relations, improved problem-solving abilities of HWs Results useful for policymakers Future evaluation of impact on health services utilisation needed	Methodology not well described Solutions, no clear action plans Relationships between different power levels in the workshops not discussed	24
Onyango-Ouma et al. (2001).^33^ An Evaluation of Health Workers for Change in Seven Settings: a useful management and health system development tool	Longitudinal implementation research, mixed methods Qualitative (key informant interviews, group interviews, FGDs) and quantitative (structured observations, time flow studies) Study done in 7 settings, 5 counties– Nigeria, Ghana, Tanzania, Kenya and Argentina	Identify impact of the HWFC interventions 3 levels of intervention explored—the community, the health facility and the district heath system	3 TPs used to identify impact Positive impact in client–provider relations, improved teamwork within a facility noticed Many changes between T1 and T3–overall wait time reduced; polite interactions between HWs and clients in all but one country, improved medicine availability and patient privacy Areas unimproved were beyond the facility (e.g. lack of water and electricity) Improvements in supervision, budgeting and drug supply were observed at the facility level	Triangulation of methods and data from different levels (clients, providers, system), strengthens the evidence of the positive impact Similarity of findings across sites gives confidence in findings Improvements in patient–provider and provider–provider relations, better working conditions for HWs and increased demand upon systemic level for response to health facilities issues	Small sample size in some sites No mention of how data were analysed, which could impede the replicability of the study	22
Ratcliffe et al. (2016).^35^ Mitigating disrespect and abuse during childbirth in Tanzania: an exploratory study of the effects of two facility-based interventions in a large public hospital	25 HW in urban facility Quantitative 3 phase design Baseline postpartum interviews and re-interviews, structured observations and interviews Intervention: ‘Respectful Maternity Care Workshops (HWFC)’ and ‘Open Birth Days’, implemented over 7 mo Evaluation: pre- and post-surveys, monthly monitoring of action plan	To describe the process of selecting and implementing a package of interventions to address disrespect and abuse of women in a health facility and preliminary outcome of the interventions on patients, providers and facility	Results from Respectful maternity care (HWFC) workshops: (1) high level of participant engagement and interaction, with suggested municipal scale up; (2) unified action plan involving management, integrated facility-wide processes Improved culture of respect noticed HWs described increased sense of autonomy, improved perceptions of supervisors and improved staff relationships Patient satisfaction with care increased markedly	Recommendations to advance knowledge about effective and efficient ways to promote respectful maternity care, more studies to be conducted, facilitating interpretation of findings and replication and scale-up of effective interventions PR strongly recommended to identify acceptable, sustainable and appropriate interventions	Not clear why senior and junior HW were separated in the workshops Implementation and evaluation phases of study overlapped; therefore, results may have been impacted by knowing an assessment was ongoing The researchers were active partners in part of the intervention. Effect of this unknown No statistical tests to measure impact	30
Webber et al. (2018).^36^ Promoting respectful maternity care in rural Tanzania: nurses' experiences of the ‘Health Workers for Change’ programme	Implementation of HWFC workshops followed by survey and qualitative FGDs 60 nurses working at hospitals, health centres and dispensaries from 6 locations	A pilot study to improve nurses’ attitudes toward pregnant women, preparatory to a project to improve their access to antenatal care and delivery facilities	Participants appreciated the training: many asked for workshops to be repeated to sustain positive changes and to include more staff HWs acknowledged their poor QoC, including negative attitudes toward women patients The workshops stimulated suggestions for improving women's experiences in future	Useful for eliciting problems among HWs and increasing self-reflection Relevant for different levels of hierarchy Practical suggestions made for incentivising women to visit facility—e.g. pay CHWs for bringing women; greater extension of services to rural areas	Incomplete methods described Lacks analysis of HWFC processes, preventing an in-depth understanding of findings	22

Abbreviations: CHW, community health worker; FGD, focus group discussion; HW, health worker; KII, key informant interview; PR, participatory research; QoC, quality of care; TP, time point.

### Outcomes of included studies

- Our analysis involved measuring changes in health workers’ motivations toward improved QoC, whether in their knowledge and skills, attitudes or behaviour. Several indicators of health workers’ motivation have been identified in the research literature, key among them job satisfaction, organisational commitment, work conscientiousness and commitment to community,^36^ all of which broadly frame the outcome indicators included in this review. We extracted the impact indicators from the included articles, expressed as specific intentions or behaviours linked to improved QoC. These nine indicators, and the number of studies in which they were included, were as follows:
- Health workers acknowledge that they sometimes treat clients badly (all studies).
- Health workers state intention to improve QoC (all studies).
- Evidence that QoC improved postintervention (six studies).^28^,^30–32^,^34,35^
- Health workers distinguish between things they could change and those beyond their control (seven studies).^18–24^
- Request to repeat or expand intervention (six studies).^28^,^30–32^,^34,35^
- Evidence of reduced interpersonal conflict in study facilities postintervention (four studies).^30–32^,^34^
- Evidence of improved health worker–client relations (four studies).^30–32^,^34^
- Evidence of reduced client waiting time (three studies).^30,32,34^
- Evidence of improved client satisfaction (three studies).^30,32,34^

We report on indicators in 15 locations, including 6 from the individual papers,^28,30,31^,^33–35^ 4 from Fonn et al.^29^ and 5 from Onyango-Ouma et al.^32^ The latter paper also includes a summary of two studies that are reported on in more detail separately, those from Kenya^20^ and Argentina,^21^ but their indicators are included only once, as reported in the individual papers.^20,21^

Our effectiveness indicators, numbered in the order presented in Table 3, are briefly summarised below:

1. Health workers admit that they sometimes treat clients badly

**Table 3.** Indicators of effectiveness of HWFC in motivating change in included studies

	Indicator of change postintervention								
Study and country	1. HWs admit they sometimes treat clients badly	2. HWs state intention to improve QoC	3. Evidence that QoC improved	4. HWs can distinguish locus of power in health system	5. HWs request that intervention be repeated or expanded	6. Evidence of reduced inter-personal conflict in facility/ies	7. Evidence of improved HW–client relations	8. Evidence of reduced client waiting time	9. Evidence of improved client satisfaction
1. Fonn and Xaba (2001).^29^ South Africa	♦	♦	♦	♦ *	♦				
2. Fonn et al. (2001).^30^									
Zambia	♦	♦		♦	♦				
Mozambique	♦	♦		♦	♦				
Senegal	♦	♦		♦	♦				
Uganda	♦	♦		♦	♦				
3. Onyango-Ouma et al. (2001).^31^ Kenya	♦	♦	♦	♦	♦	♦	♦	♦	♦
4. Pittman, Blatt and Rodriguez (2001).^32^ Argentina	♦	♦	♦	♦**	♦	↓	♦		♦
5. Onyango-et al. (2001)^33^									
Nigeria (Kwara)	♦	♦	♦	♦	♦	♦	♦	♦	♦
Nigeria (Kaduna)	♦	♦	♦	♦	♦	♦	♦	♦	♦
Tanzania (Ilala)			♦		♦	♦	♦	♦	♦
Tanzania (Kinondoni)			♦		♦	♦	**✦**	♦	♦
Ghana					♦	♦			
6. Shaikh et al. (2006).^34^ Pakistan	♦	♦		♦					
7. Ratcliffe et al. (2016).^35^ Tanzania	♦	♦	♦	♦	♦	♦	♦		♦
8. Webber et al. (2018).^36^ Tanzania	♦	♦	♦		♦				

♦Indicator was measured and positive changes occurred.

↓Indicator was measured and negative changes occurred.

*The paper does not specifically report on this indicator, but Table 1 indicates awareness of these differences in levels and where motivation to higher authorities is needed.

**This desire was stronger at T2 than at T3 because HWs had become frustrated with lack of receptiveness from supervisor. They did not feel confident in their ability to effect change or improve.

✦Indicator measured but no impact was found.

In all the study sites, health workers acknowledged that they sometimes treated their clients poorly and that such clients perceived services offered as uncaring and punitive. They attributed their behaviour to a lack of positive role models,^28^ having to work late for deliveries,^28^ late or no salaries,^28,29^ equipment shortages^29,32^ and lack of supervisory support.^28^ In such cases their insensitive behaviour was a mechanism for coping with other problems.^29^

2. Health workers state their intention to improve QoC

Once health workers acknowledged their insensitive behaviour and its causes, they also said they could improve their performance and, in 12 sites, stated their intention to do so. Illustrations of improvements they planned to make were arriving at work on time,^30^ showing respect for others, including vulnerable clients,^28,29^ and encouraging women to report earlier for services.^35^ In Argentina, staff developed strategies to persuade men to attend the clinic so that they could better relate to their wives’ concerns.^31^

3. Evidence that QoC improved

In 9 of the 10 sites in which this indicator was measured, positive changes in the services resulted, such as the introduction of regular staff meetings,^28,31,32^ a conflict resolution workshop^28^ and a strategy for relating to superiors in South Africa,^28^ the creation of a cost-sharing fund to obtain stock-out drugs from the private sector and a kitchen garden to amplify healthy food for in-patients in Kenya,^30^ as well as an increased appreciation of patient rights among both providers and clients in Tanzania.^34^

4. Health workers can distinguish the locus of power in the health system

The ability of health workers to distinguish changes they could make themselves from those beyond their authority resulted from the workshops in 11 of the 15 sites. This was an indicator that resulted from the intervention, reflecting an important step in taking responsibility for one's actions. In Tanzania,^35^ for example, the action plan developed at the end of the workshops focused on activities the health facility could conduct mainly with their own resources. In the four-country study in Africa,^19^ suggestions emerged for how health workers themselves could improve QoC or motivate others to do so, delineating those areas where higher level support was needed. In most sites health workers also identified obstacles they were unable to address and recommended that their training be expanded to incorporate these issues. A major concern across most sites was a lack of drugs and equipment,^29,33^ which was generally beyond health workers’ power to control.^29,33,35^

5. Health workers request that intervention be repeated or expanded

The spontaneous request from workshop participants to repeat or expand the intervention was reported in all but one site. This was a finding that emerged from the intervention in most studies and is included as an impact indicator because it demonstrates the enthusiasm and motivational power generated by HWFC. In Tanzania, participants suggested expanding the workshops to a wider group of staff, municipalities and health system levels, with more involvement of higher-level management to facilitate and sustain positive changes.^34^ In Argentina, authorities said they planned to incorporate participatory methods more often into in-service training following the intervention.^31^

6. Evidence of reduced interpersonal conflicts in study facilities

Interpersonal conflicts in the study health facilities were measured in eight sites postintervention and a decrease was observed in seven sites. In several facilities the initiation of regular staff meetings stimulated by HWFC provided an opportunity for interpersonal differences to be aired and tackled as a team. In Argentina, by contrast, health staff motivation to address this issue declined over time, attributed to a continuing negativity from the director of the study clinic.^21^ Despite this, the value of the intervention was recognised at higher levels in the health system, resulting in several positive changes.

7. Evidence of improved health worker–client relations

This indicator was measured in seven sites and showed a positive impact in six of them.^22^ Observational data confirmed improvements in providers’ attitudes and client sensitivity, such as becoming less punitive and more responsive to patient needs. In Ghana, however, no evidence of change was documented.

8. Evidence of reduced client waiting time

Client waiting time in facilities was measured through observation in five sites at three time points and important reductions were found in all facilities. For example, in Kenya, average client waits declined from baseline to 9 mo after the intervention by 34% (41 min),^30^ in Tanzania (Kinondoni) by 39%,^32^ and in Nigeria (Kaduna) by 53% in the antenatal unit.^32^ The time saved depended upon the particular unit observed and whether the length of waiting time preintervention was excessively long, such as in Kenya, where it was 2 h on average.^32^ Clients in most sites also observed this reduction and reported that they felt their time in the services was better spent.^32^

9. Evidence of improved client satisfaction

Satisfaction among clients preintervention and postintervention was assessed in eight sites and positive results were seen in seven. In Tanzania, where the intervention was used to mitigate disrespect during childbirth, there was a marked increase in patient satisfaction.^35^ In Kenya, clients reported that providers were more sympathetic^30^ and less likely to rebuke them, and that nurses were more willing to accept babies who had been delivered at home.^32^ They also mentioned that drug availability had increased, due to the cost-sharing fund that was created by the health workers mentioned above.^30^ In Kaduna, clients noted that staff were more likely to greet them and that they felt more confident in asking questions.^32^

### Overall impact of HWFC

The impact of HWFC was positive and remarkably similar across the varied sites. While not all indicators were measured in each study, those included showed positive impacts overall. There was wide recognition among health staff that QoC was poor and needed to be improved and there was evidence of actual changes postintervention as a result of action plans developed by the health staff themselves. Health workers also learned to separate out those actions that were in their own power to control, as opposed to others that needed to be taken at higher levels. There was also a spontaneous expression of satisfaction with the workshops, and a wish to see them repeated and even expanded. Changes in interpersonal relationships among peers were also evident, although it declined in one setting.^31^ This was not unexpected, as the participatory approach allowed for the gradual revelation of negative sentiments that may have been suppressed at baseline, but were later revealed through the application of the intervention, allowing for their eventual resolution. Indicators of the impact of HWFC on client experiences was also positive, where measured. Overall, positive interpersonal relationships with clients measurably improved, and waiting times were reduced in all five sites where they were measured. Evidence of client satisfaction was found in seven of the eight sites where it was assessed.

## Discussion

The results of this review confirm the importance of participatory approaches for motivating positive change among health workers in LMICs, in particular HWFC. They also demonstrate that, apart from HWFC, very few other studies have focused on healthcare providers’ own experience of the constraints they face in their daily work and/or suggested interventions to address them.^37^ While other participatory methodologies where the researchers are part of the problem-solving process may also produce meaningful outcomes, we maintain that the power imbalances so created could inhibit health workers’ responses.

The HWFC approach was found to be applicable, with a minimal amount of adaptation, in very different settings, from small and medium-sized rural health facilities to large urban hospitals, in Africa, Asia and Latin America. Its flexibility in accommodating the incorporation of unique, culturally appropriate content into the workshop series was also demonstrated. Further, although originally targeted at the issue of poor female client–health worker interactions, it proved useful in uncovering a host of challenges faced by health workers in their working environments, often resulting in negative coping strategies when health workers felt incapable of resolving them.

The importance of supervisory support as a key determinant of employee motivation and job satisfaction in LMICs has been widely recognised and is arguably the most significant challenge health workers face.^10,38^ Data from our review substantiates this observation: across all studies such support was found to be critical to health workers’ motivation and outcomes. Where supervisors were open to their suggestions, changes were often implemented. Where they were not, the momentum generated by the intervention diminished, leading to frustration and further demoralisation. In this respect, healthcare management interventions, such as regular supportive and constructive supervision visits^39^ and feedback to staff on job performance^4,40^ may be promising approaches when delivered in synergy with health worker-led participatory interventions such as HWFC.^41,42^

### Conclusion

This systematic review has shown that motivational interventions for health personnel in LMICs that are truly participatory can be an important catalyst toward universal health, emphasising the renewal and strengthening of the first level of care, as well as country ownership. In fact, HWFC is what ownership looks like: a locally appropriate methodology; implementation in familiar health service settings; focus on the self-identified needs of health provider and cultivating health providers’ own suggestions for tackling them.^43^ Local guidelines are more likely to be implemented because they consider factors such as these. Thus, several of the reviewed studies recommended that HWFC be mainstreamed into the training curricula of health workers and repeated intermittently to sustain its momentum and build on emergent action plans. Participatory research should be an integral part of any such process. Although cost-effectiveness of the intervention was not assessed, it requires few additional resources beyond health workers’ time and an outside facilitator and can also be incorporated within the schedules of health facilities.

While HWFC has been shown to be a useful intervention in motivating health workers to improve their performance, it also demonstrated once again that they face daily challenges in their work and that these cannot be addressed by health teams alone. Nor can the impact of a single intervention be expected to last, unless reinforced by other supportive measures from higher levels of the health system. Systemic approaches to address the improved motivation and performance of health teams, from managers to local levels, is urgently needed.^4^

While not a focus of this study, there is ample evidence of poor QoC in high-income countries^44^ as well as in LMICs.^45^ More than ever, COVID-19 is threatening the well-being and safety of health workers worldwide, who are also facing tremendous workplace challenges and burnout. Their cry for help in the face of overburdened health systems is real and present. Finding ways to listen and respond to their needs, identify coping strategies and propose solutions and mitigation measures through participatory methodologies, such as HWFC, could well pay off in terms of increased job satisfaction and long-term retention.

## Supplementary Material

ihac070_Supplemental_File

## Data Availability

The data that support the findings of this systematic reviews are available from the corresponding author, [OO], upon request.

## Footnotes

1 The issue of poor provider–client relationships has also been described in high-income countries^46^ (albeit to a lesser extent), and often manifested differently as a result of different health system structures, access to information and support mechanisms, legislation, etc. For this reason, the literature from high-income countries was also consulted, although not systematically, for comparative purposes, and in case of encountering any promising participatory research approaches not found in the LMIC literature.

2 This period was selected because it dates from the development of the HWFC methodology in the late 1990s.

3 Health Workers for Choice, Health Workers for Teens: a manual to improve the quality of care for teenage clients and Kenya community health workers manual.^21–23^

4 Submitted as Supplementary Table(s).

5 Several studies reported on a broad methodology, the *Heshima* project, for reducing mistreatment and abuse of women during delivery in Africa but the separate effects on health workers were not teased out. However, while not included in the strict systematic review, they were considered in our overall analysis.^47^

6 Now incorporated into the Gender and Health Division of the School of Public Health at the University of Witwaterstrand, Johannesburg.
