# Supplementary material for: A systematic review of participatory approaches to empower health workers in low- and middle-income countries, highlighting Health Workers for Change
Source: Int Health. 2022 Nov 9;15(4):462–73. doi: 10.1093/inthealth/ihac070 (PMC10318970; doi:10.1093/inthealth/ihac070)
Supplement: ihac070_Supplemental_File [file ihac070_supplemental_file.docx]

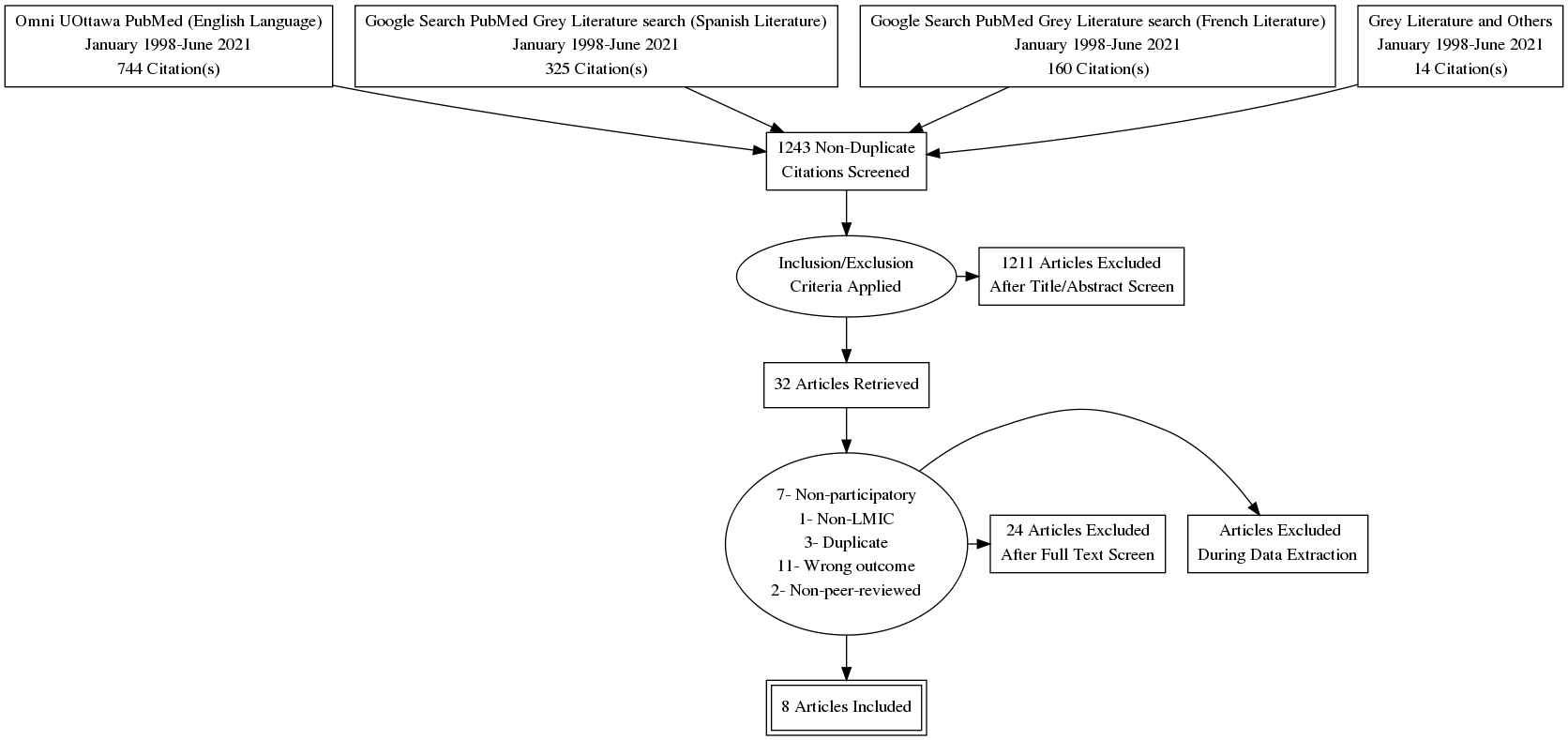


FIGURE1: PRISMA Flowchart

The PRISMA flowchart shows data sources and search results. 1243 titles resulted from the initial search. Following tile/abstract review, 32 articles were selected for full review. An additional 24 articles were excluded leaving eight included in the analysis.

**SUPPLEMENTARY TABLE 1: STUDIES INCLUDED FOR FULL TEXT REVIEW – REASONS FOR INCLUSION OR EXCLUSION**

| Title | Original peer-reviewed research | Uses participatory empowerment/  training methods for health workers | Adequately describes results | Approach in motivating positive change evaluated | Comments | Decision re Inclusion in SR |
| --- | --- | --- | --- | --- | --- | --- |
| 1. Promoting respectful maternity care in rural Tanzania: nurses' experiences of the "Health Workers for Change" program (Webber, Chirangi, & Magatti, 2018)^21^ | *Yes* | *Yes* | *Yes* | *Yes* | Part of an implementation research in Tanzania.  Used 6 workshops  Survey and FGD after training to evaluate results | *Include* |
| 2. Health providers’ opinions on provider–client relations: results of a multi-country study to test Health Workers for Change (Fonn et al., 2001)^18^ | *Yes* | *No – it reviews existing results* | *Yes* | *Yes* | Focused on only rural areas.  Describes acceptability for African Countries  Summarizes outcome of the workshops & impact measured were data available | *Include* |
| 3. Health Workers for Change: developing the initiative (Fonn & Xaba, 2001)^17^ | *Yes* | *Yes* | *Partly* | *No – only action plan described* | Describes first study of the methodology in South Africa rural clinic | *Include* |
| 4. Health Workers for Change as a health systems management and development tool (Vlassoff & Fonn, 2001) | *Yes* | *No – it reviews existing results* | *No* | *No* | Discusses the insights behind the HWFC methodology | *Exclude* |
| 5. An evaluation of Health Workers for Change in seven settings: a useful management and health system development tool (Onyango-Ouma et al., 2001) ^14^ | *Yes* | *Yes* | *Yes* | *Yes* | *Details on results 2-3 time points for 7 countries* | *Include*  *Assign JN as 2nd reviewer* |
| 6. Health Workers for Change: a tool for promoting behaviour change among health providers (Shaikh, Rabbani & Rahim,2006)^19^ | *Yes* | *Yes* | *Yes* | *Yes* | *Describes applicability of HWFC in Pakistan* | *Include* |
| 7. Introducing Health Workers for Change: from transformation theory to health systems in developing countries (Haaland & Vlassoff, 2001) | *Yes* | *No – introductory article to Special Issue* | *Yes* | *No* | *Summarizes the approach to be presented in the subsequent papers* | *Exclude* |
| 8. Women’s Health Leadership to Enhance Community Health Workers as Change Agents (Ingram et al., 2016) | *Yes* | *No* | *Yes* | *Yes* | *Not participatory*  *Not LMIC* | *Exclude* |
| 9. The Health Workers for Change impact study in Kenya (Onyango-Ouma, Thiong’o, Odero & Houma, 2001)^15^ | *Yes* | *Yes* | *Yes* | *Yes* | *Detailed results at 3 time points* | ***Include*** |
| 10. An assessment of the impact of Health Workers for Change in Avellaneda, Province of Buenos Aires, Argentina (Pittman, Blatt & Rodriguez, 2001)^16^ | *Yes* | *Yes* | *Yes* | *Yes* | *Detailed results at 3 time points* | *Include* |
| 11. An iterative process for training design and implementation increased health workers' knowledge for taking nutrition behavior change to scale (Gonzalez et al., 2019) | *Yes* | *no – more training to increase knowledge* | *Yes* | *No* | *Knowledge production oriented rather than participatory method to motivate broad behaviour change among health workers* | *Exclude* |
| 12. ‘It makes you someone who changes with the times’: health worker and client perspectives on a smartphone-based counselling application deployed in rural Tanzania (Hackett et al., 2019) | *Yes* | *No* | *No* | *No* | *Not relevant to participatory methodology to change behaviour* | *Exclude* |
| 13. Commitment to Change Among Health Care Workers in Pakistan (Bakari, Hunjra & Jaros, 2020) | *Yes* | *No - describes a scale to measure commitment to change* | *No* | *No* | *Aims to evaluate C2C and its use in but not to motivate broad behaviour change among health workers* | *Exclude* |
| 14. The impact of social norms interventions on clinical behaviour change among health workers: protocol for a systematic review and meta-analysis (Cotterill et al., 2019) | *Yes* | *No* | *No – not yet completed* | *No* | *Describes a planned SR –registered with Prospero* | *Exclude* |
| 15. Front-line health workers: leading the frontiers of change (Dyall, 2012) | *Not clear* | *Partly* | *No– not yet completed* | *Not clear* | *Approach in New Zealand for Maori front-line HW involvement/ empowerment (though not LMIC, could be included perhaps because Maori) but impact not yet studied* | *Exclude* |
| 16. Community health workers and social change: An introduction (Maes, 2015) | *Yes* | *No* | *Yes, as a literature review* | *Partially* | *Participatory methodology not discussed, results insufficient* | *Exclude* |
| 17. The relationship of psychological empowerment and readiness for organizational changes in health workers, Lorestan, Iran (Khammarnia, Ravandgard & Asadi, 2014) | *Yes* | *No* | *Yes* | *No* | *Interviews only*  *Not participatory*  *Prospective, not actual changes* | *Exclude* |
| 18. Support workers as agents for health behavior change: An Australian study of the perceptions of clients with complex needs, support workers, and care coordinators (Lawn et al., 2016) | *Yes* | *Yes, but limited to role of support workers (assistants, carers & volunteers)* | *Yes* | *Yes* | *Not LMIC but showed how participatory training methods could be effective* | *Exclude but perhaps useful for overall conclusions* |
| 19. Motivation, comportements organisationnels discrétionnaires et bien-être en milieu Africain: Quand le devoir oblige? (Levesque, Blais, Hess, & Sulsky, 2004) | *yes* | *no* | *yes* | *Partly, but only through questionnaire* | *Based solely on questionnaire responses regarding elements of motivation* | *Exclude but perhaps useful for overall conclusions* |
| 20. Dimension humaine du management logistique : stress, fatigue et santé des salariés (Large & Paché, 2016) | *yes* | *no* | *yes* | *Partly* | *Very broad focus, discusses salary issues extensively, not very relevant to LMIC health worker motivation* | *Exclude* |
| 21. Évaluation du rendement et motivation au travail: propositions de recherche pour une rétroaction sur le rendement qui favorise la satisfaction des besoins psychologiques fondamentaux (Grenier, Chiocchio & Beaulieu, 2012) | *yes* | *no* | *no* | *no* | *Goes deeply into theory and is not empirical* | *Exclude* |
| 22. Motivations des Agents Communautaires: Approche multi-facette de MAHEFA pour soutenir la motivation des AC Community-Based Integrated Health Program (USAID, MAHEFA, JSI, ND) | *no - 15 technical briefs on 5-year intervention program* | *Partly – some participatory methods* | *Not in detail but good summary* | *yes* | *Not peer reviewed -*  *grey literature & a mix of interventions, lack of detail* | *Exclude* |
| 23. Tutoría virtual para profesionales de enfermería noveles (Fortuño et al., 2013) | *yes* | *yes, but virtual* | *yes* | *yes* | *Not LMIC Mainly describes training of nursing students – still shows the value of a motivational, supportive approach* | *Exclude but perhaps useful for overall conclusions* |
| 24. Servicio de humanismo y bioetica de la Fundacion Santa Fe de Bogota, Colombia Dos anos de experiencia (Prieto, 2017) | *Yes, describes a process but not its impact* | *partly* | *partly* | *No. only described* | *No impact evaluation* | *Exclude* |
| 25. Motivación del Equipo Básico de Atención Integral en Salud como factor que contribuye al logro de los objetivos del nuevo Modelo de Atención Integral (Montoya & Garcia, 2001) | *yes* | *no – interviews only* | *yes* | *no* | *Interesting study of motivational elements among staff in a new community model of care.* | *Exclude but perhaps*  *useful for background or discussion* |
| 26. Retos para enfermería en el cuidado de personas con dolor: una forma de humanización (Montealegre Gómez, 2014) | *yes* | *no – mainly reflective plus literature review on pain* | *yes* | *no* | *Interesting literature review & reflections of author of motivation & training of nurses in pain management* | *Exclude* |
| 27. Repensando las prácticas de salud y educación en las complejidades sociales. Descripción y análisis de una metodología de investigación acción participativa (Corvalàn, 2013) | *yes* | *No, not clear what the methods were – only that researcher spent time in the communities. Interviews poorly described.* | *no, very selective, not systematically presented* | *partly, based on participatory action research, but not clearly focused on health workers* | *Interesting because of use of Freire methods but poorly described and not evaluated.* | *Exclude* |
| 28. *Projecte* RIU: cambios percibidos por agentes de salud y profesionales tras una intervención en salud en un barrio en situación de vulnerabilidad (Aviñó, et al., 2014) | *yes* | *yes* | *partly, but intervention not well described. One has to read about it elsewhere and try to discren which part of the intervention is being referred to* | *partly but approach not well described* | *High income country (Spain)*  *Paper does not go deeply enough into linking specific interventions with outcomes.* | *Exclude but perhaps useful for introduction/discussion* |
| 29.  MCSP Nigeria HWCF brief | *Not peer reviewed* | *Yes* | *Yes* | *Yes* | *Include*  *Shows results more qualitative* | *Exclude Not peer reviewed, can be referenced in the introductions.*  *Can include only for introduction, but not for results* |
| 30. Mitigating disrespect and abuse during  childbirth in Tanzania: an exploratory study  of the effects of two facility-based  interventions in a large public hospital (Ratcliffe et al., 2016)^20^ | *Yes* | *Yes* | *Yes* |  | *Detailed discusses 3-time points results* | *Include* |
| 31. Health workers for choice. Working to improve quality of abortion services (Varkey, Fonn & Ketlhapile, ND | *No* | *Yes* | *No* | *No – manual format* | *Manual as opposed to results* | *Exclude but perhaps useful for discussion* |
| 3 *Hea* 32. Health Worker for Teens: A Manual to Improve the Quality of Care for Teenage Clients, 2015 (RMCH, UKAID, CARMMA, ND) | *No, grey literature PDF format* | *Yes* | *No* | *No – manual format* | *Manual as opposed to results* | *Exclude but perhaps useful for discussion* |

**SUPPLEMENTARY TABLE 2A - CRITICAL APPRAISAL TOOL FOR INCLUDED PAPERS**

(Scoring – Good =4, Fair =3, Poor =2, Very Poor =1 (Possible total of 32)

Suggest that if score is 15 or less we exclude from SR even if other criteria met

Reviewer 1 score in Black

Reviewer 2 score in Red font

Author, date, and title: Webber, Chirangi, B., Magatti, N. (2018); Promoting respectful maternity care in rural Tanzania: nurses' experiences of the "Health Workers for Change" program

Date: July 2021

| Article section | Good | Fair | Poor | Very poor | Comment | Agreed Score |
| --- | --- | --- | --- | --- | --- | --- |
| Introduction and aims | 4  4 |  |  |  | Good background and relevant literature to introduce aim of study. Literature from other African countries quoted – Ethiopia, Kenya. Called out different types of abuse done to women receiving pregnancy-related care. Aimed at improving care providers attitude towards pregnant women. | 4 |
| Method and data |  | 3  3 |  |  | Sample of questions asked not described in methods section. Method appropriate for the objective of the study. | 3 |
| Sampling |  |  | 2  2 |  | Locations considered and number of participants mentioned. No mention of how the sampling size was determined. Gender distribution slightly skewed, although more women are more common is such occupations. How the 60 participants were chosen was not clearly described to exclude selection bias. | 2 |
| Data analysis |  |  | 2  2 |  | No description of actual analysis. On | 2 |
| Ethics and bias |  | 3  3 |  |  |  | 3 |
| Findings/results |  | 3  3 |  |  | Only analysed results on providers not on facility or patient | 3 |
| Transferability/generalizability |  |  | 2  2 |  | Small population and only in one country, not representative of other LMICs | 2 |
| Implications and usefulness |  | 3 | 2 |  |  | 3 |

Agreed: 22/32

**SUPPLEMENTARY TABLE 2B - CRITICAL APPRAISAL TOOL FOR INCLUDED PAPERS**

Author, date, and title: _ S FONN,1 AS MTONGA,2 HC NKOLOMA,3 G BANTEBYA KYOMUHENDO,3 L DASILVA,4 E KAZILIMANI,4 S DAVIS5 AND R DIA (2001) Health providers’ opinions on provider–client relations: results of a multi-country study to test *Health Workers for Change*

Date: July 2021

| Article section | Good | Fair | Poor | Very poor | Comment | Agreed |
| --- | --- | --- | --- | --- | --- | --- |
| Introduction and aims |  | 3  3 |  |  | Clear introduction and aim to roll out the HWFC intervention to a larger population after initial pilot. | 3 |
| Method and data | 4  4 |  |  |  | Focused on rural communities and had multiple centers | 4 |
| Sampling | 4 | 3 |  |  | In one country, all employees were included to participate in the intervention. Though women still more, some sites had a slightly less skewed gender distribution. Multiple countries with similar rural settings | 3 |
| Data analysis |  |  | 2  2 |  | Summarized | 2 |
| Ethics and bias |  | 3 | 2 |  |  | 3 |
| Findings/results |  | 3 | 2 |  |  | 3 |
| Transferability/generalizability |  | 3 | 2 |  | Methodology is generalizable but no actual long-term impact/result. | 3 |
| Implications and usefulness | 4 | 3 |  |  |  | 4 |

Agreed: 25/32

**SUPPLEMENTARY TABLE 2C - CRITICAL APPRAISAL TOOL FOR INCLUDED PAPERS**

Author, date, and title: SHARON FONN AND MAKHOSAZANA XABA Health Workers for Change: developing the initiative

Date: July 2021

| Article section | Good | Fair | Poor | Very poor | Comment | Agreed |
| --- | --- | --- | --- | --- | --- | --- |
| Introduction and aims | 4 | 3 |  |  | Aim to identify provider perception at problem posing | 4 |
| Method and data |  |  | 2  2 |  | Role Play, Questionnaires, Group Discussions were done. Not clear how they were analysed. Using the 6 HWFC workshops. Qualitative data, wasn’t clearly | 2 |
| Sampling |  | 3  3 |  |  | 16 participants from varying from doctors, nurses, cleaners. The ratio of engagement not mentioned. Gender ration not mentioned though, most providers were female in an earlier introduction. Not clear how many participants attended all 6 workshops. Participants attended different sessions as they were available. | 3 |
| Data analysis |  |  | 2 | 1 | Not clear description of analysis | 1 |
| Ethics and bias |  | 3 | 2 |  | Brief mention of gender-related issues as part of the workshop, though only mentioned in regard to workshop participants. No mention of ethical or bias among researchers. No mention of consent obtained from study participants | 2 |
| Findings/results | 4  4 |  |  |  | Qualitative – result of each workshop clearly described. Achieved the aim of problem solving, by identifying potential solutions from the providers themselves | 4 |
| Transferability/generalizability |  | 3  3 |  |  | Provides some context | 3 |
| Implications and usefulness |  | 3  3 |  |  | Contributes evidence on the usefulness of the HWFC intervention | 3 |

Agreed: 22/32

**SUPPLEMENTARY TABLE 2D - CRITICAL APPRAISAL TOOL FOR INCLUDED PAPERS**

Author, date, and title: WASHINGTON ONYANGO-OUMA,1 ROSE LAISSER,2 MUSIBA MBILIMA,3 MARGARET ARAOYE,4 PATRICIA PITTMAN,5 IRENE AGYEPONG,6 MAIRO ZAKARI,7 SHARON FONN,8 MARCEL TANNER9 AND CAROL VLASSOFF10 An evaluation of Health Workers for Change in seven settings: a useful management and health system development tool

Date: July 2021

| Article section | Good | Fair | Poor | Very poor | Comment | Agreed |
| --- | --- | --- | --- | --- | --- | --- |
| Introduction and aims | 4 |  | 2 |  |  | 3 |
| Method and data | 4  4 |  |  |  | Similar health centers were chosen across all countries. Centers with diverse service provisions and which were regularly/frequently used by the community. | 4 |
| Sampling | 4 | 3 |  |  | Most centers but not all meant the characteristic of high patient ratio with wide range of services These criteria were met in the case of the  studies in Tanzania, Kenya and in Nigeria-Kaduna. In  Nigeria-Kwara, the daily patient load at both the General  Hospital and the PHC facilities appeared to be low | 3 |
| Data analysis | 4 |  |  | 1 | Qualitative and Quantitative data collected and analysed | 1 |
| Ethics and bias | 4 |  |  | 1 | Relevant health authorities were approached by the researched prior to commencement of study except in Ghana where the approach was informal | 1 |
| Findings/results | 4 | 3 |  |  |  | 4 |
| Transferability/generalizability |  | 3  3 |  |  |  | 3 |
| Implications and usefulness | 4 | 3 |  |  |  | 3 |

Agreed: 22/32

**SUPPLEMENTARY TABLE 2E - CRITICAL APPRAISAL TOOL FOR INCLUDED PAPERS**

Author, date, and title: __ Health Workers for Change: a tool for promoting behaviour change among health providers B.T. Shaikh, F. Rabbani and M. Rahim **m**ay-July 2006

Date: July 2021

| Article section | Good | Fair | Poor | Very poor | Comment | Agreed |
| --- | --- | --- | --- | --- | --- | --- |
| Introduction and aims | 4  4 |  |  |  | Clear background of the HS | 4 |
| Method and data |  | 3  3 |  |  |  | 3 |
| Sampling |  |  | 2 | 1 |  | 1 |
| Data analysis |  |  | 2 | 1 | No clear description | 1 |
| Ethics and bias | 4 | 3 |  |  |  | 4 |
| Findings/results | 4 | 3 |  |  |  | 4 |
| Transferability/generalizability |  | 3 | 2 |  |  | 3 |
| Implications and usefulness |  | 3 | 2 |  |  | 3 |

Agreed: 24/32

**SUPPLEMENTARY TABLE 2F - CRITICAL APPRAISAL TOOL FOR INCLUDED PAPERS**

Author, date, and title: The Health Workers for Change impact study in Kenya WASHINGTONONYANGO-OUMA,1 FREDERICKWTHIONG’O,2 THERESAMAODERO3ANDJOHNHOUMA

Date: July 2021

| Article section | Good | Fair | Poor | Very poor | Comment | Agreed |
| --- | --- | --- | --- | --- | --- | --- |
| Introduction and aims | 4  4 |  |  |  |  | 4 |
| Method and data | 4  4 |  |  |  | involved collecting data from clients, health facility staff and  health managers at the system level at baseline (T1), at 4  weeks after the intervention (T2) and at 9 months after the  intervention (T3) | 4 |
| Sampling |  | 3  3 |  |  | Just one center | 3 |
| Data analysis |  |  | 2  2 |  | Qualitative and quantitative, were. No detail of type of variable and analysis performed  collected at three levels: client, facility and system | 2 |
| Ethics and bias |  |  | 2  2 |  | Not clearly discussed | 2 |
| Findings/results | 4  4 |  |  |  |  | 4 |
| Transferability/generalizability |  | 3  3 |  |  | Only done in rural setting, may not apply to urban settings | 3 |
| Implications and usefulness | 4  4 |  |  |  |  | 4 |

Agreed: 26/32

**SUPPLEMENTARY TABLE 2G - CRITICAL APPRAISAL TOOL FOR INCLUDED PAPERS**

Author, date, and title: An assessment of the impact of Health Workers for Change in Avellaneda, Province of Buenos Aires, Argentina PATRICIA PITTMAN,1 GRACIELA BLATT2 AND PATRICIA RODRIGUEZ3

Date: July 2021

| Article section | Good | Fair | Poor | Very poor | Comment | Agree |
| --- | --- | --- | --- | --- | --- | --- |
| Introduction and aims | 4 | 3 |  |  |  | 3 |
| Method and data |  | 3  3 |  |  |  | 3 |
| Sampling |  | 3  3 |  |  | Only morning shift staff were included in intervention | 3 |
| Data analysis |  |  | 2  2 |  |  | 2 |
| Ethics and bias |  | 3  3 |  |  |  | 3 |
| Findings/results | 4  4 |  |  |  |  | 3 |
| Transferability/generalizability |  | 3  3 |  |  |  | 3 |
| Implications and usefulness |  | 3  3 |  |  |  | 4 |

Agreed: 25/32

**SUPPLEMENTARY TABLE 2H - CRITICAL APPRAISAL TOOL FOR INCLUDED PAPERS**

Author, date, and title: Corvalàn, Repensando las prácticas de salud y educación en las complejidades sociales. Descripción y análisis de ua metodología de investigación acción participative

Date: July 2021

| Article section | Good | Fair | Poor | Very poor | Comment | Agreed |
| --- | --- | --- | --- | --- | --- | --- |
| Introduction and aims |  |  | 2  2 |  |  | 2 |
| Method and data |  |  | 2  2 |  | semi-structured interviews  were carried out over a period of five months and were articulated with  were articulated with observations of the neighborhood's residence and schools.  schools in the neighborhood | 2 |
| Sampling |  |  | 2  2 |  | 30 families in their homes were selected, How?? | 2 |
| Data analysis |  |  | 2  2 |  |  | 2 |
| Ethics and bias |  |  | 2  2 |  |  | 2 |
| Findings/results |  |  | 2  2 |  |  | 2 |
| Transferability/generalizability |  |  | 2 | 1 |  | 1 |
| Implications and usefulness |  |  | 2  2 |  |  | 2 |

Agreed: 15/32

**SUPPLEMENTARY TABLE 2I - CRITICAL APPRAISAL TOOL FOR INCLUDED PAPERS**

Author, date, and title: Mitigating disrespect and abuse during childbirth in Tanzania: an exploratory study of the effects of two facility-based interventions in a large public hospital Hannah L. Ratcliffe1,2*, David Sando1,3, Goodluck Willey Lyatuu3, Faida Emil3, Mary Mwanyika-Sando4, Guerino Chalamilla3,4ˆ, Ana Langer1 and Kathleen P. McDonald1,5

Date: July 2021

| Article section | Good | Fair | Poor | Very poor | Comment | Agreed |
| --- | --- | --- | --- | --- | --- | --- |
| Introduction and aims | 4  4 |  |  |  | Clear aims to reduce disrespect and abuse and improve maternal care | 4 |
| Method and data | 4 | 3 |  |  | Pre- & Post- design. Questionnaires, Structured interviews, Direct observations of provider-patient interaction. Conducted between January 2013 and  December 2014. 3 phases of the study, baseline, intervention, and post intervention. Baseline facility date on birth outcomes was collected. 5 groups of 15-20 participants each completed all 6 workshops. | 3 |
| Sampling | 4  4 |  |  |  | 88 staff where approached, 76 agreed to participate.  For the direct observation. Random sampling of every second woman who came to register was interviewed. | 4 |
| Data analysis | 4  4 |  |  |  | Data analysis was performed using STATA Version 13 for example, very details explanation of all variables was clearly outlined | 4 |
| Ethics and bias | 4 | 3 |  |  |  | 4 |
| Findings/results | 4  4 |  |  |  | Qualitative and Quantitative results well described for different time points. | 4 |
| Transferability/generalizability |  | 3  4 |  |  |  | 3 |
| Implications and usefulness | 4  4 |  |  |  | Very useful for | 4 |

Agreed: 30/32
